# PYCR1 Downregulation Induces Autophagy Dependent Apoptosis Through Inhibiting PI3K/AKT/mTOR Axis in Human Hepatocellular Carcinoma Cells

**DOI:** 10.1155/ancp/3662809

**Published:** 2026-02-23

**Authors:** Junli Zhang, Yachao Hou, Xinxin Jin, Biao Gu, Wenjuan Wu

**Affiliations:** ^1^ Department of Blood Transfusion, Bengbu Third People’s Hospital Affiliated to Bengbu Medical University, Bengbu, 233030, Anhui, China; ^2^ Bengbu Medical University Key Laboratory of Cancer Research and Clinical Laboratory Diagnosis, Bengbu Medical University, Bengbu, 233030, Anhui, China, bbmc.edu.cn; ^3^ Department of Biochemistry and Molecular Biology, School of Laboratory Medicine, Bengbu Medical University, Bengbu, 233030, Anhui, China, bbmc.edu.cn

**Keywords:** apoptosis, autophagy, hepatocellular carcinoma, pyrroline-5-carboxylate reductase 1

## Abstract

**Background:**

Hepatocellular carcinoma (HCC) is a global malignant tumor type. Pyrroline‐5‐carboxylate reductase 1 (PYCR1) is a metabolic enzyme that exhibits pro‐tumor properties in cancer progression. However, the exact molecular mechanism of PYCR1 in HCC progression is still unclear.

**Methods:**

CCK8, EdU, and Transwell assays were used to measure the proliferation, migration, and invasion of HCC cells, respectively. Immunostaining and flow cytometry were used to detect cellular autophagy and apoptosis.

**Results:**

The downregulation of PYCR1 can inhibit the survival, proliferation, migration, and invasion of HCC cells. At the same time, downregulation of PYCR1 induces autophagy and subsequently activates cell apoptosis. Therefore, we pretreated HCC cells with mTOR activators or inhibitors to inhibit or promote autophagy, leading to an inhibition or an increase in apoptosis. Simultaneously, the PI3K activators or inhibitors to activate or inhibit the PI3K/AKT/mTOR pathway also lead to inhibition or activation of autophagy and apoptosis.

**Conclusion:**

The downregulation of PYCR1 induces autophagy‐dependent apoptosis in HCC cells by inhibiting the PI3K/AKT/mTOR pathway, revealing a novel mechanistic link in HCC pathophysiology.

## 1. Introduction

There are ~900,000 new cases of hepatocellular carcinoma (HCC) worldwide every year, and it is third in cancer‐related mortality, with about 800,000 deaths per year and an increasing trend [1]. Despite concerted efforts in the diagnosis, treatment, and molecular characterization of HCC, the overall 5‐year survival rate of patients is inferior to 30%, with most having a poor prognosis [2, 3]. The high mortality rate and poor prognosis are primarily related to the diagnosis of many patients at advanced stages, which lack effective treatment options [4]. Recurrence and distant metastasis in advanced HCC are the primary reasons for treatment failure [5]. Therefore, identifying new diagnostic and therapeutic targets is an imperative research goal.

Autophagy is a programed intracellular degradation process. Cells form autophagosomes by encapsulating degradation products and transporting them to lysosomes for digestion to meet metabolic needs, update organelles, maintain cellular homeostasis, and perform apoptosis and autophagy [6, 7]. Research has found that autophagy promotes the activation of cell apoptosis, leading to cell death. Some proteins in the autophagy pathway also promote cell apoptosis, activating apoptosis during the development of autophagosomes at the beginning of autophagy [8, 9]. In bladder cancer, apoptosis induced by pentacyclic triterpenoids can be attenuated or eliminated by autophagy inhibitors or ATG7 silencing with targeted siRNA [10]. In addition, the compounds activate autophagy‐dependent apoptosis through the AMPK/mTOR/ULK1 pathway [11]. Meanwhile, the PI3K/AKT/mTOR signaling pathway induces tumorigenesis through various mechanisms and is involved in cellular autophagy, such as 3‐MA (3‐methyladenine, an enzyme PI3K inhibitor necessary for autophagosome formation), which can inhibit autophagy [11, 12]. It also can inhibit cell apoptosis, such as by suppressing the conformational changes of Bax and phosphorylating other apoptotic structural components such as Bax and Caspases9 at the mitochondrial level, and by downregulating the expression of tumor suppressor protein p53 in the nucleus [13, 14]. Nevertheless, the mechanism of autophagy‐dependent apoptosis mutual regulation is still unknown.

Pyrroline‐5‐carboxylate reductase 1 (PYCR1) is a key enzyme involved in energy metabolism [15]. It is responsible for the second step of synthesizing proline from glutamate. While generating proline, PYCR1 participates in energy metabolism and regulates key physiological processes such as cell cycle, autophagy, and apoptosis in mitochondria [16–18]. Recently, PYCR1 has received attention as an essential regulatory factor in cancer development [19–21]. It functions as a tumor promoter, and high expression in colon adenocarcinoma is associated with poor prognosis [20]. PYCR1 silencing enhances the anti‐tumor effect of the chemotherapy drug 5‐fluorouracil (5‐FU) [20]. Downregulation of PYCR1 can obstruct glycolysis and lessen histone lactylation. PYCR1 is a key regulatory factor affecting tumor cell metabolism and gene expression in HCC progression [17]. Knockout of the PYCR1 gene inhibits growth and functions of Hep‐3B and HepG2 cells, promotes cell apoptosis, and G1 blockade [22]. However, research on its function and autophagy‐dependent apoptosis is limited. This study aims to elucidate the carcinogenic effects and corresponding molecular mechanisms of PYCR1 and reveal its potential as a therapeutic target for HCC.

## 2. Methods and Materials

### 2.1. Cell Culture and Transfection

LO2, SMMC‐7721, HUH7, SNU‐449, and Hep‐3B (CAS, Shanghai, China) cell lines were cultured in RPMI‐1640 (SNU‐449, LO2, SMMC‐7721) or DMEM (Hep‐3B, HUH7) with 10% FBS (Gibco, USA) in a cell incubator. Using the Lipofectamine 2000 transfection kit to achieve siRNA transfection into cells. The sequences are as follows: siRNA1, 5′‐CUAACUUGCUCUCGAUCCUTT−3′(sense); siRNA2, 5′‐GCCCACAAGAUAAUGGCUATT‐3′(sense); and siRNA3, 5′GAAGAAGCUGUCAGCGUUUTT −3′(sense).

### 2.2. Reagents and Antibody

LY294002 (LY), 740Y‐P, Rapamycin (RAP), MHY1485 (MHY), and Z‐VAD‐FMK (ZVF) also were brought from MCE (Shanghai, China). Antibodies were purchased as follows: Primary antibodies against p‐ULK1 (1:1000), ULK1 (1:1000), P62 (1:1000), Beclin1 (1:1000), Bcl‐2 (1:2000), Bax (1:2000), Caspase‐3 (1:100), PARP (1:3000), PYCR1 (1:1000), and β‐actin (1:5000), and secondary antibodies (1:5000) were obtained from Proteintech (Chicago, IL). LC3B (1:1000), Caspase‐9 (1:100), p‐PI3K (1:1000), PI3K (1:1000), AKT1 (1:1000), p‐AKT1 (1:2000), P‐mTOR (1:1000), and mTOR (1:2000) were obtained from Cell Signaling Technology (Danvers, MA, USA).

### 2.3. Bioinformatic Analyses

Analyze the expression and related indicators of PYCR1 using GEPIA (http://gepia.cancer-pku.cn/. accessed 15 March 2024) and the Human Protein Atlas (https://www.proteinatlas.org/), and evaluate the prognostic role of PYCR1 using the KM plotter database (http://kmplot.com/analysis/. accessed 15 March 2024).

### 2.4. Cell Proliferation Assay

Collect cells (1000 cells/well), inoculate them with 96 well plates, and wait for 24 h for growth. Then, add 10 µl CCK‐8 solutions into cells at 37°C for 2 h. The results were detected every 24 h.

### 2.5. EdU Proliferation Assay

The cell was handled for 2 days and collected. Cell proliferation was assessed using an EdU kit. All fluorescent images were investigated employing a Zeiss fluorescence microscope.

### 2.6. Wound Healing Assay

When the number of cells occupies 90% of the area of the 6‐well plate, use a pipette tip to draw lines on the cells to create a linear wound. Wash the culture plate with PBS to remove floating cells. After 24 h of cell growth, photos of the wound were taken using a light microscope (Olympus, USA).

### 2.7. Transwell Assay

Take the transfer (Corning) chamber with or without matrix gel, add 200 µl serum‐free cell suspension into the upper chamber, and add 800 µl culture medium containing 10% FBS into the lower chamber. After 24 h of cell growth, observe the number of cells penetrating the chamber membrane under a microscope and take photos.

### 2.8. TUNEL Assay

One‐step TUNEL staining is being used to measure the apoptosis of cells. In short, after treatment with cell crawling, 4% paraformaldehyde solution fixation, and 0.3% Triton X‐100 permeability, HCC cells were tagged with fluorescein‐12‐dUTP, and the nuclei were spotted with 4,6‐diamino‐2‐phenylindole (DAP). Take photographs utilizing a Zeiss fluorescence microscope.

### 2.9. Flow Cytometry Assay

Collect the experimental cells after 48 h of growth, stain them with Annexin V/PI in the dark for 30 min to obtain labeled cells, and measure the apoptosis rate using a flow cytometer (Cytomics FC500).

### 2.10. Transmission Electronical Microscopy (TEM)

Experimental cells were fixed overnight at 4°C using 2.5% glutaraldehyde. Subsequently, the samples were post‐fixed with 1% osmium tetroxide, dehydrated, and sectioned into ultra‐thin slices (50–60 nm) via ultramicrotomy. Following staining with 1% uranyl acetate and lead citrate, the sections were examined under a TEM, and images were captured at magnifications of 10,000× and 25,000×.

### 2.11. Immunofluorescence

Cells were seeded onto 35 mm Petri dishes, washed with PBS, fixed at room temperature with 4% paraformaldehyde for 30 min, and dripped with 0.5% Triton X‐100 for membrane permeation treatment for 15 min. After adding 10% BSA and blocking at room temperature for 2 h, incubated with a rabbit anti‐LC3‐I/II. Subsequently, samples were treated with a goat anti‐rabbit IgG AF488. Nuclei were counterstained with DAPI (2 min), and images were acquired using a laser scanning confocal microscope to analyze LC3‐I/II fluorescence.

### 2.12. RNA Extraction and Quantitative Real‐Time PCR

After total RNA extraction, it is reverse transcribed into cDNA. Quantitative PCR was conducted, with each reaction run in triplicate. The primers were as follows: PYCR1, 5′‐TCCATTGAGAAGAAGCTGTCAG‐3′ and 5′‐CATCAATCAGGTCCTCTTCC AC‐3,′; and β‐actin, 5′‐GTGGACATCCGCAAAGAC‐3′ and 5′‐AAAGGGTGTAACGC AACTA‐3′.

### 2.13. Protein Extraction and Western Blotting

Cells in each individual group were collected, and total proteins were extracted using RIPA buffer (Beyotime, China) and denatured at 95°C. The 40 µg of BCA kit quantified protein per well was subjected to electrophoresis and membrane transfer. Subsequently, the blocked membranes were then probed with primary antibodies during an overnight incubation at 4°C. The second antibody was incubated for 2 h. Finally, the gel imaging system was exposed.

### 2.14. Statistical Analysis

All data are expressed as mean ± standard deviation (SD). For comparisons among multiple groups, statistical analysis was performed using one‐way ANOVA with Tukey’s test in SPSS 20.0. Statistical significance was set at *p* < 0.05.

## 3. Result

### 3.1. PYCR1 is Upregulated in HCC, and Its High Expression is Associated With Patient Poor Prognosis

According to GEPIA database results, PYCR1 mRNA showed marked upregulation in HCC tissues (Figure 1A). Using the HPA database to obtain immunohistochemical results of HCC patients, PYCR1 showed significantly higher protein expression levels than adjacent tissues (Figure 1B). Furthermore, both the GEPIA database and Kaplan–Meier plotter analyses indicated PYCR1 overexpression as a negative prognostic marker in HCC (Figure 1C, D). Among five HCC cell lines examined, the results revealed that PYCR1 expression substantially exceeded LO2 normal hepatocyte levels, and SNU‐449 and Hep‐3B had relatively high expression (Figure 1E, G), which were selected for subsequent experiments. In addition, the silencing efficiency of PYCR1 after cell transfection with small interfering RNA was detected, and the results showed that the mRNA and protein levels of siRNA1, siRNA2, and siRNA3 were significantly reduced, with siRNA2 showing the best silencing efficiency (Figure 1F, H).

Figure 1PYCR1 is overexpressed in HCC and correlates with poor prognosis. (A, C) Transcript per million and overall survival of PYCR1 in HCC Tumor (*n* = 369) compared to normal corresponding group (*n* = 160) from GEPIA database analysis; (B) protein‐level validation from Human Protein Atlas confirmed PYCR1 overexpression in HCC specimens; (D) Kaplan–Meier analysis demonstrated that high PYCR1 expression predicted worse clinical outcomes, including overall survival (OS), progression‐free survival (PFS), and disease‐specific survival (DSS); (E, G) western blot and RT‐qPCR assays showing protein and mRNA levels of PYCR1 in five HCC cell lines(Gels/Blots are sheared); and (F, H) successful PYCR1 knockdown was confirmed in siRNA‐transfected cells, with significant reduction observed at both protein and transcript levels,  ^∗^ *p* < 0.05,  ^∗∗^ *p* < 0.01,  ^∗∗∗^ *p* < 0.001.

**Figure:**
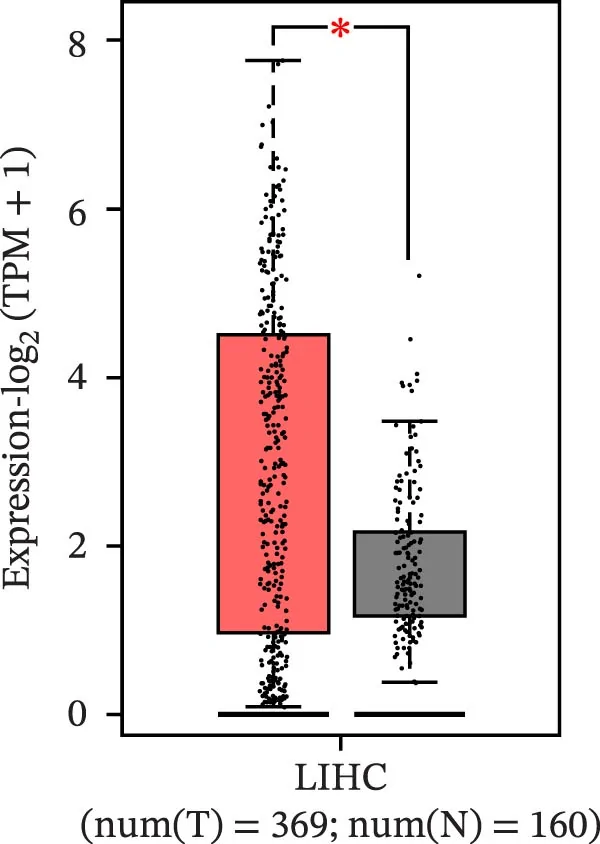
(A)

**Figure:**
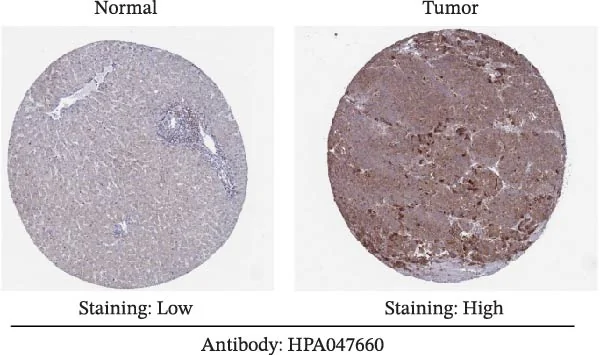
(B)

**Figure:**
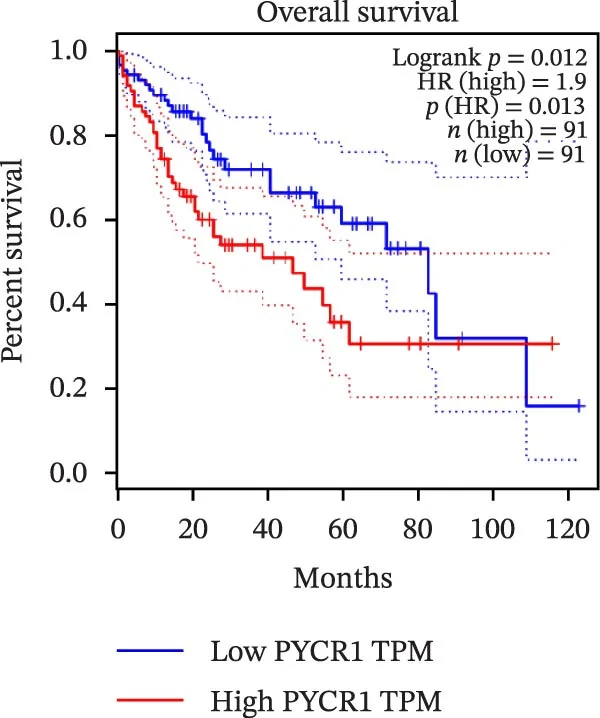
(C)

**Figure:**
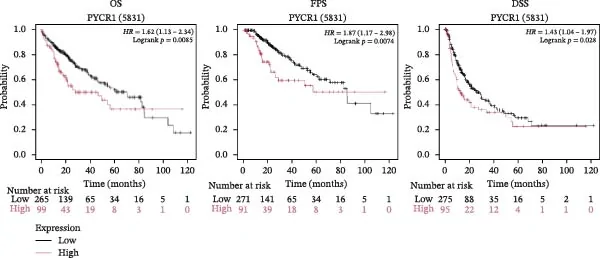
(D)

**Figure:**
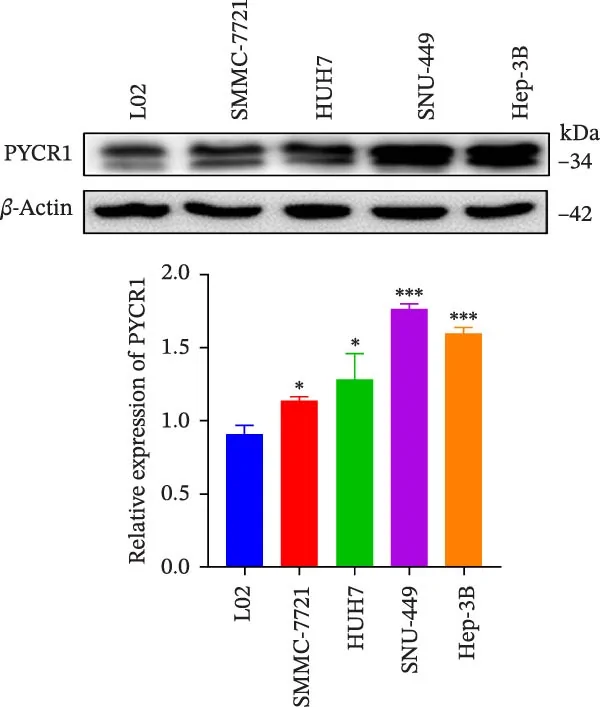
(E)

**Figure:**
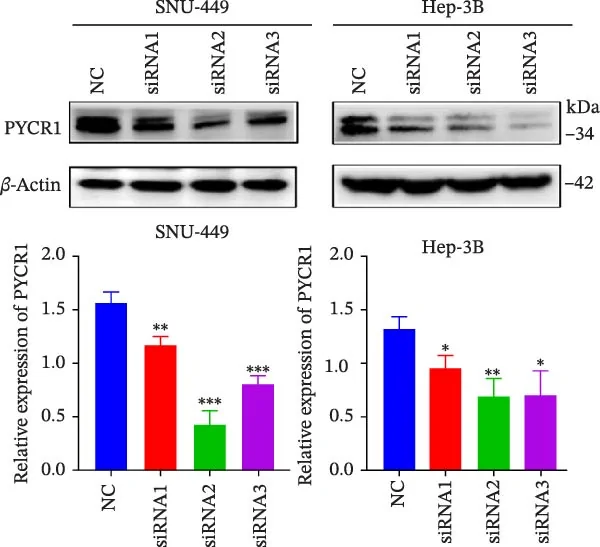
(F)

**Figure:**
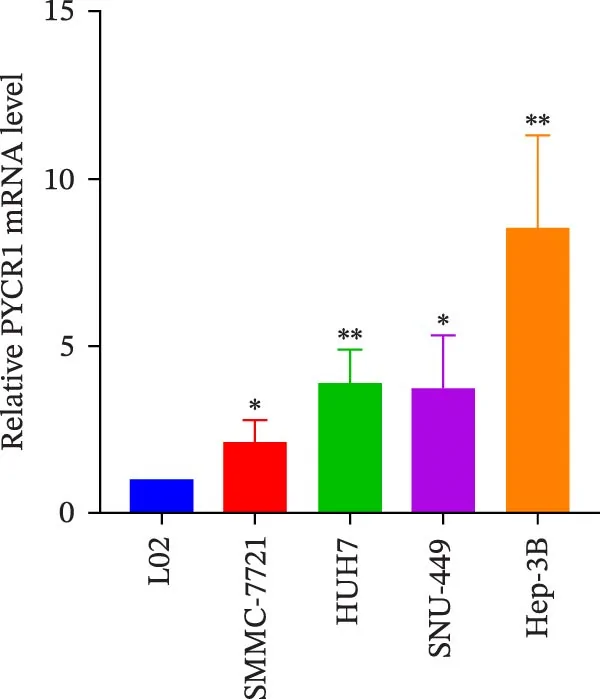
(G)

**Figure:**
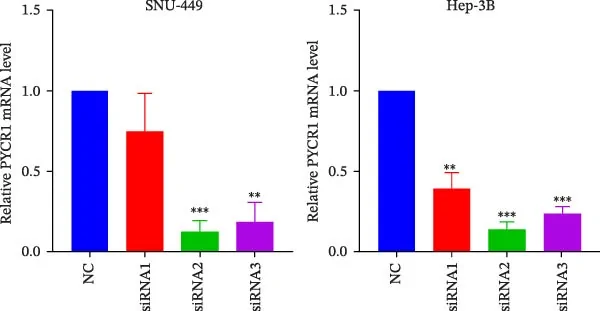
(H)

### 3.2. Downregulation of PYCR1 Suppresses HCC Cells Growth and Metastasis in Vitro

The impact of PYCR1 on HCC cells was evaluated through systematic in vitro functional assays. Experiments related to cell growth include CCK8 and EdU staining analysis. Based on the absorbance data measured by the CCK8 experiment, the cell proliferation curve was plotted to show that after downregulating PYCR1, the cells’ proliferative capacity significantly decreased, and the inhibitory impact became more pronounced with time in HCC (Figure 2A). Additionally, EdU staining results also showed that silencing PYCR1 remarkably decreased both EdU‐positive cell counts and proliferation rates (Figure 2B). Collectively, these data establish PYCR1 as a critical regulator of HCC cell growth. Next, we subsequently examined its role in tumor cell metastasis. A wound‐healing test showed that HCC cells downregulated by PYCR1 had a larger wound area and a significantly smaller cell migration area compared to the control group (Figure 2C). Silencing PYCR1 in HCC cells markedly reduced cellular migration, as evidenced by the decreased number of cells traversing the Transwell chamber membrane (Figure 2D). These results preliminarily indicate that downregulation of PYCR1 significantly reduces the metastatic ability of HCC cells.

Figure 2Downregulation of PYCR1 suppresses HCC cell growth and metastasis in vitro. (A) Cell proliferative capacity was measured by Cell Counting Kit‐8 assay in PYCR1 downregulated SNU‐449 and Hep‐3B cells; (B) EdU staining assays were performed to evaluate the cellular proliferation in PYCR1 downregulated SNU‐449 and Hep‐3B cells—red fluorescence indicates EDU staining, while blue fluorescence indicates DAPI staining; (C) wound‐healing assays were used to determine the cell migration ability of HCC cells with downregulated PYCR1; and (D) transwell assays were used to determine the migration and invasion abilities of HCC cells with downregulated PYCR1.  ^∗^ *p* < 0.05,  ^∗∗^ *p* < 0.01,  ^∗∗∗^ *p* < 0.001.

**Figure:**
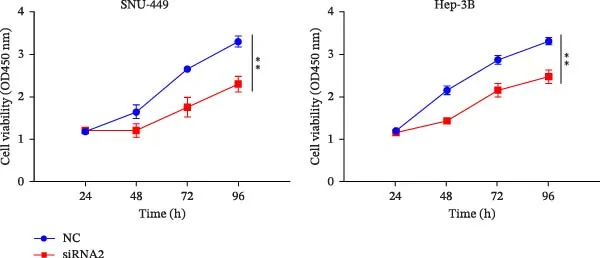
(A)

**Figure:**
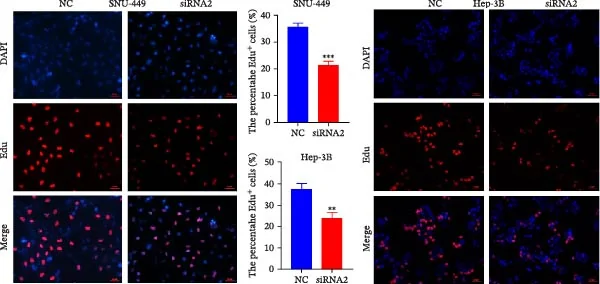
(B)

**Figure:**
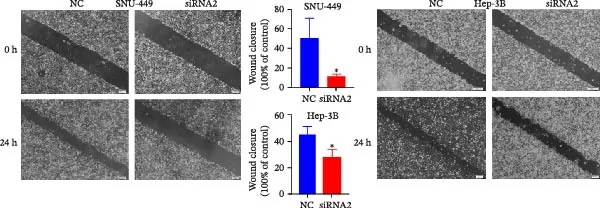
(C)

**Figure:**
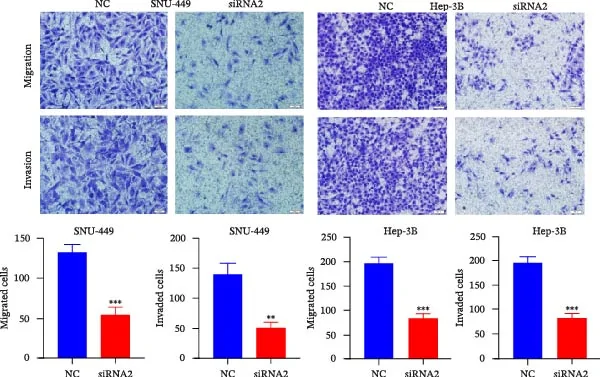
(D)

### 3.3. Downregulation of PYCR1 Induced Apoptosis in SNU‐449 and Hep‐3B Cells

To assess the role of PYCR1 in apoptosis, Annexin V‐FITC/PI staining was performed to measure apoptotic cell populations. Silencing PYCR1 markedly elevated apoptosis rates, with early and late apoptosis levels reaching 17.3% and 26.8%, respectively, significantly exceeding those of the control group (Figure 3A). Consistent with these observations, TUNEL staining was performed on apoptotic cells, and it was found that there was a pronounced rise in TUNEL‐positive cells upon PYCR1 suppression (Figure 3B, C). Further mechanistic analysis via Western blotting indicated that PYCR1 downregulation altered the expression of key apoptotic regulators, such as Bax/Bcl‐2, PARP/cleaved‐PARP, Caspase9/cleaved‐caspase9, etc. (Figure 3D, E). In addition, the use of apoptosis inhibitor ZVF further confirmed that downregulation of PYCR1 leads to cell apoptosis. Western blotting showed that ZVF can inhibit the upregulation of protein expression such as Bax/Bcl‐2, PARP/cleaved‐PARP, and Caspase9/cleaved‐caspase9 caused by downregulation of PYCR1, thereby inhibiting cell apoptosis caused by downregulation of PYCR1 (Figure 3F–H).

Figure 3Downregulation of PYCR1‐induced apoptosis in SNU‐449 and Hep‐3B cells. (A) FCM analysis of apoptosis in SNU‐449 and Hep‐3B cells following PYCR1 downregulation; (B, C) apoptosis was detected using TUNEL assay. Scale bar, 50 µm; and (D–H) immunoblot analysis and quantification of apoptosis‐related protein expression in SNU‐449 and Hep‐3B cells (gels/blots are sheared).  ^∗^ *p* < 0.05,  ^∗∗^ *p* < 0.01.

**Figure:**
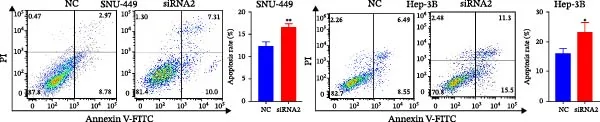
(A)

**Figure:**
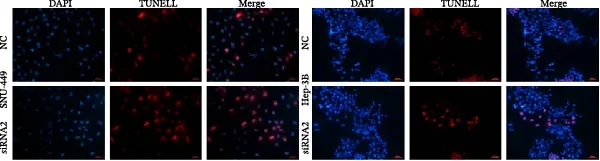
(B)

**Figure:**
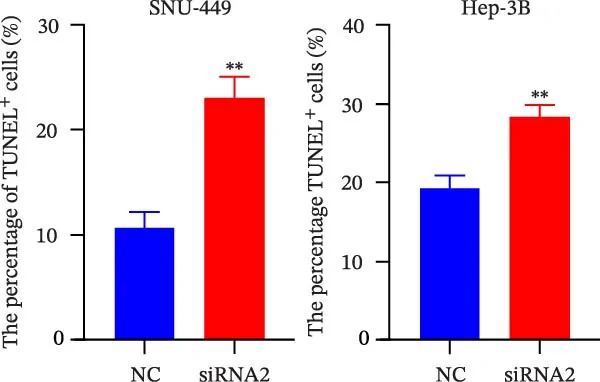
(C)

**Figure:**
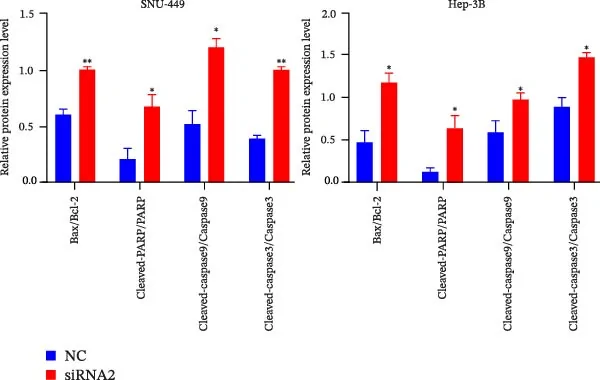
(D)

**Figure:**
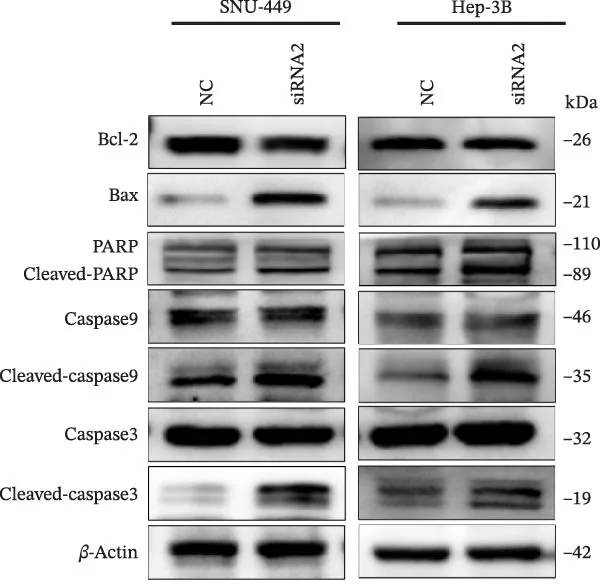
(E)

**Figure:**
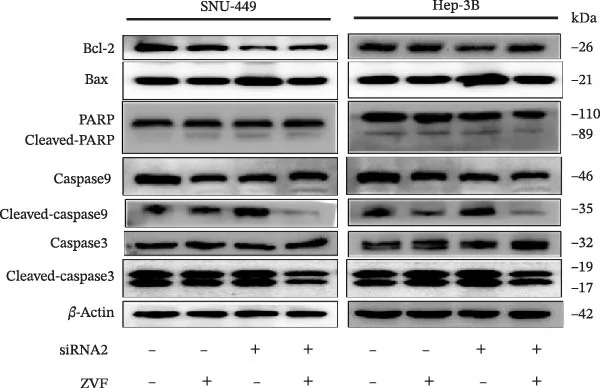
(F)

**Figure:**
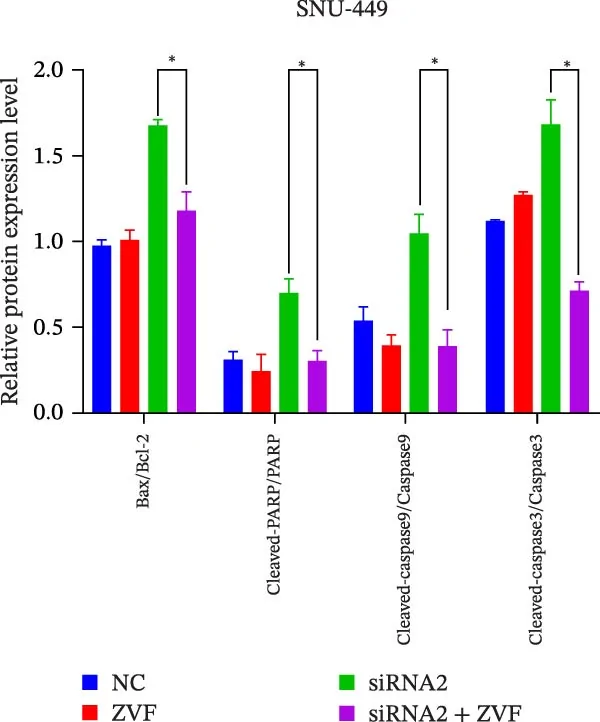
(G)

**Figure:**
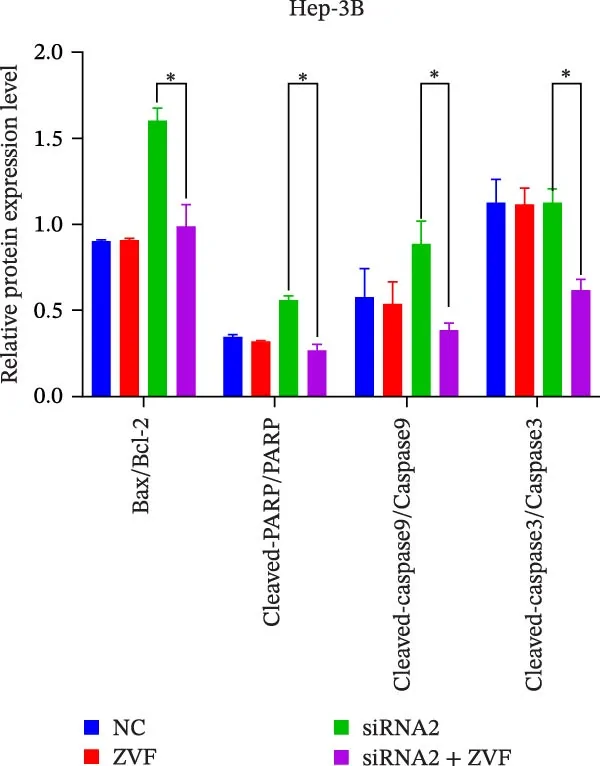
(H)

### 3.4. Downregulation of PYCR1 Induced Cells Autophagy Via Suppressed PI3K/AKT/mTOR Pathway

To determine whether PYCR1 contributes to autophagy regulation of HCC cells, we transfected cells with siRNA2 of PYCR1 for 24 h and then observed using a TEM. To evaluate the involvement of PYCR1 in autophagy, SNU‐449 and Hep‐3B cells were transfected with PYCR1 siRNA2 for 24 h and analyzed via TEM. Untreated controls exhibited minimal autophagic activity, whereas PYCR1‐silenced cells displayed a marked accumulation of double‐membrane vesicles containing electron‐dense cytoplasmic material, a hallmark of enhanced autophagy (Figure 4A, C). This observation was corroborated by immunofluorescence staining, which showed a pronounced increase in LC3 puncta formation upon PYCR1 knockdown compared to the negative control (Figure 4B, D). Further western blot analysis revealed elevated LC3‐II/I ratios and reduced p62 levels (Figure 4F, H), alongside upregulated expression of autophagy‐related proteins, including Beclin1 and p‐ULK1/ULK1 (Figure 4F, H). Collectively, these findings suggest that PYCR1 silencing disrupts autophagic flux. Mechanistically, PYCR1 suppression attenuated the PI3K/AKT/mTOR pathway, as evidenced by decreased phosphorylation of PI3K, AKT1, and mTOR, while total protein levels remained unaltered (Figure 4E, G).

Figure 4Downregulation of PYCR1‐induced cells autophagy via the suppressed PI3K/AKT/mTOR pathway. (A) Ultrastructural analysis by transmission electron microscopy (× 10,000 and × 25,000 magnification) revealed autophagic vacuoles in siRNA2‐transfected cells; (B) immunofluorescence staining demonstrated increased LC3 puncta formation in PYCR1‐deficient cells; (C, D) quantitative analysis of (A) autophagosome counts and (B) LC3 fluorescence intensity; and (E–H) western blot analysis showed altered expression of autophagy markers and PI3K/AKT/mTOR pathway components following PYCR1 silencing,  ^∗^ *p* < 0.05,  ^∗∗^ *p* < 0.01 (gels/blots are sheared).

**Figure:**
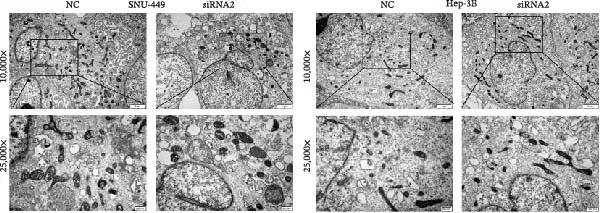
(A)

**Figure:**
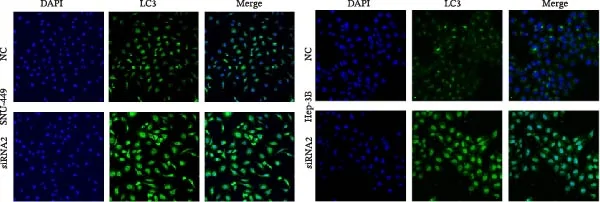
(B)

**Figure:**
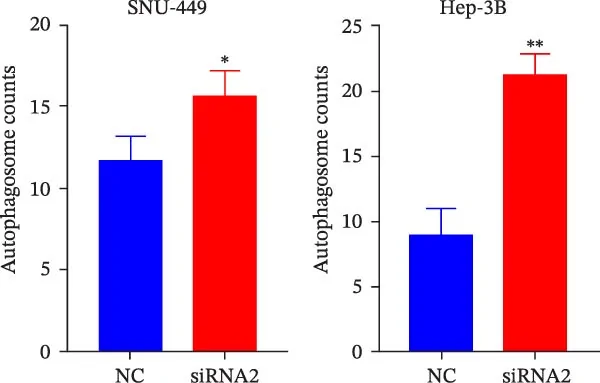
(C)

**Figure:**
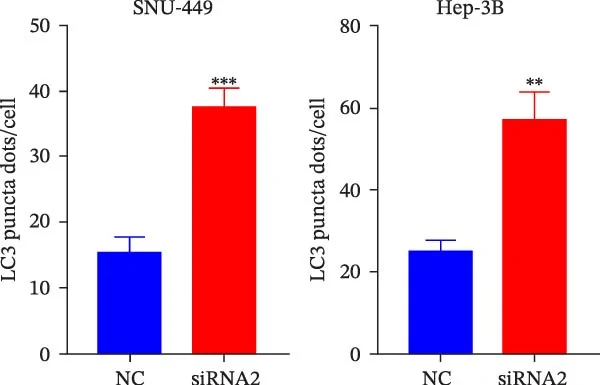
(D)

**Figure:**
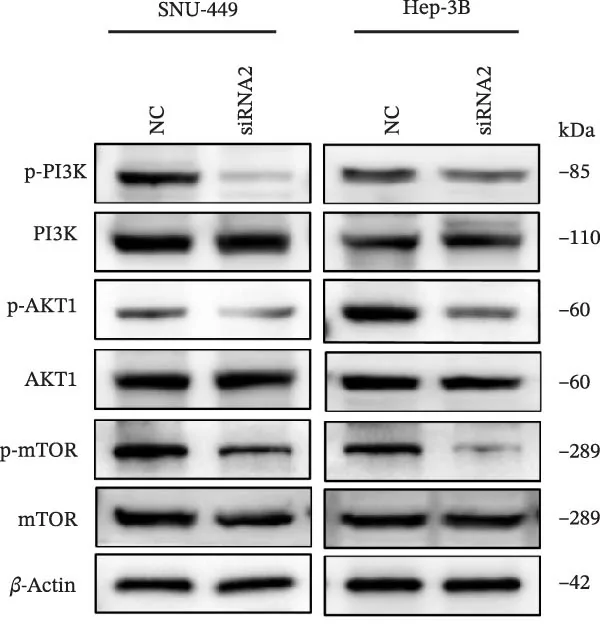
(E)

**Figure:**
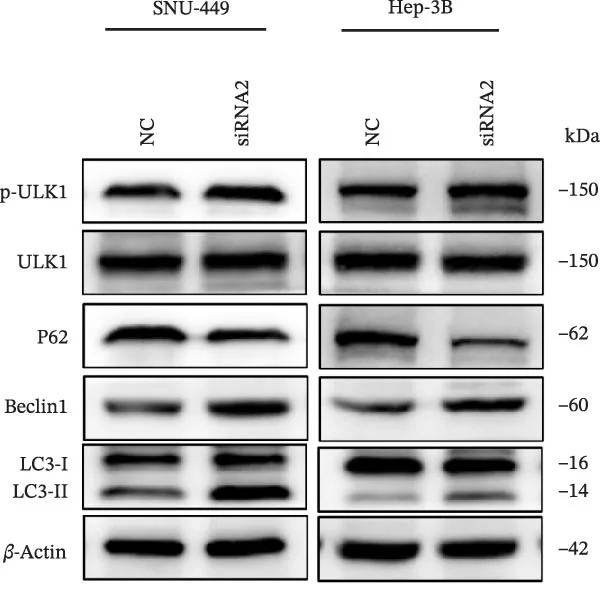
(F)

**Figure:**
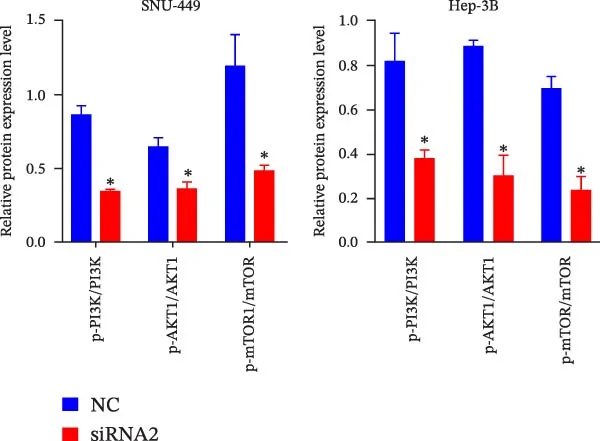
(G)

**Figure:**
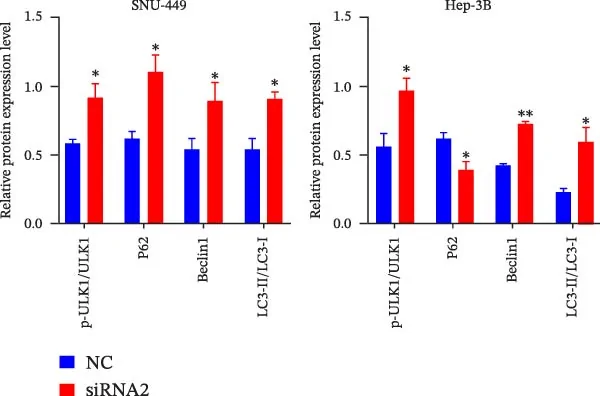
(H)

### 3.5. Downregulation of PYCR1 Induced Autophagy Dependent Apoptosis

Autophagy and apoptosis are the core mechanisms by which cells respond to stress, and there is a synergistic or antagonistic relationship between the two. To examine the interplay between autophagy and apoptosis following PYCR1 knockdown, SNU‐449 and Hep‐3B cells were subjected to RAP (rapamycin, an mTOR inhibitor that activates autophagy) or MHY (MHY1485a, an mTOR activator that inhibits autophagy) prior to PYCR1 suppression. This experimental design allowed us to assess how autophagy modulation influences apoptotic responses in the context of PYCR1 deficiency. MHY inhibits autophagy by suppressing fusion between autophagosomes and lysosomes. The findings displayed that MHY decreased the level of the LC3B‐II/I ratio, the p‐ULK1/ULK1 ratio, and the expression of Beclin1, while increasing the level of P62 (Figure 5B). Meanwhile, pharmacological inhibition of autophagy with MHY markedly altered the expression profile of key apoptotic regulators. Treatment with MHY led to significant downregulation of pro‐apoptotic proteins (cleaved caspase‐3, cleaved caspase‐9, Bax, and cleaved‐PARP) and concurrent upregulation of the anti‐apoptotic protein Bcl‐2 (Figure 5B). Moreover, in immunofluorescence and flow cytometry assays, MHY treatment of PYCR1 downregulated cells can significantly reduce the number of autophagic cells labeled with LC3 fluorescence and apoptotic cells induced by PYCR1 downregulation (Figures 5A, 6A). In contrast to MHY, RAP—an mTOR inhibitor that stimulates autophagy—exerted opposing effects on apoptotic regulation (Figures 5A, C, 6A). These findings collectively demonstrate that PYCR1 knockdown triggers apoptosis through an autophagy‐dependent mechanism.

Figure 5PYCR1 knockdown induces autophagy‐dependent apoptosis. (A) SNU‐449 and Hep‐3B cells were pretreated for 24 h with LY (10 μM), 740Y‐P (30 μM), and RAP (25 nM), or for 4 h with MHY (10 μM) and ZVF (40 μM), followed by apoptosis assessment using flow cytometry ( ^∗^ *p* < 0.05,  ^∗∗^ *p* < 0.01); (B, C) siRNA‐mediated PYCR1 knockdown cells were treated with MHY1485 (10 μM) or rapamycin (250 nM) for 4 h. Western blot analysis was performed to evaluate apoptotic markers (Bcl‐2, Bax, cleaved‐PARP, cleaved caspase‐9, and cleaved caspase‐3) and autophagy markers (LC‐3II/I ratio, p‐ULK1/ULK1, p62, and Beclin1) (gels/blots are sheared).

**Figure:**
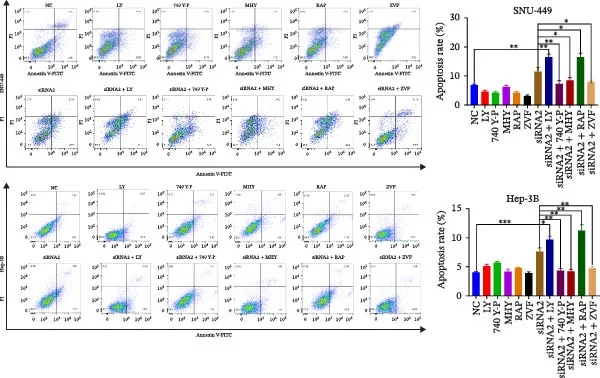
(A)

**Figure:**
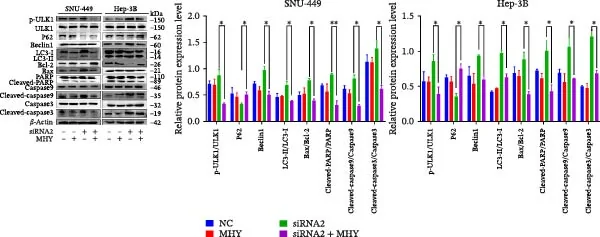
(B)

**Figure:**
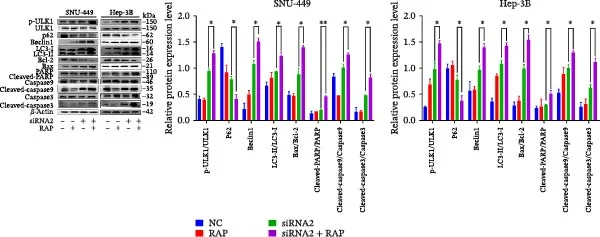
(C)

Figure 6Downregulation of PYCR1‐induced autophagy‐dependent apoptosis via the suppressed PI3K/AKT/mTOR pathway in HCC. (A) Immunofluorescence detection of autophagy marker LC3 expression in SNU‐449 and Hep‐3B cells; (B, C) Western blot assay of p‐PI3K, PI3K, AKT1, p‐AKT1, mTOR, and p‐mTOR proteins in SNU‐449 and Hep‐3B cells; and (D, E) Western blot assay of autophagy and apoptotic proteins in siRNA2‐transfected cells treated with LY or 740 Y‐P (gels/blots are sheared).

**Figure:**
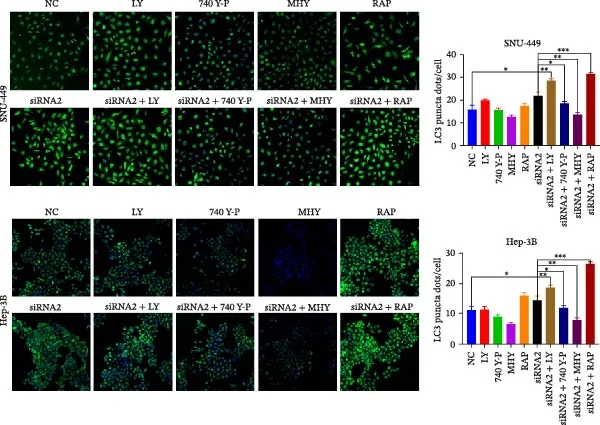
(A)

**Figure:**
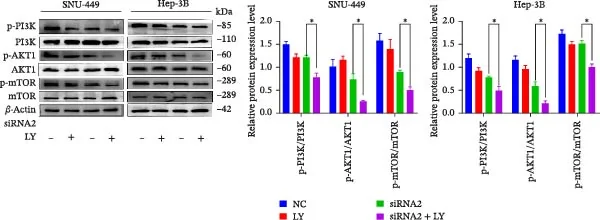
(B)

**Figure:**
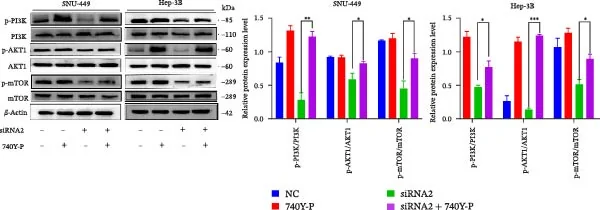
(C)

**Figure:**
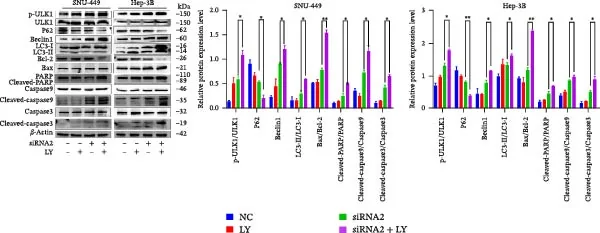
(D)

**Figure:**
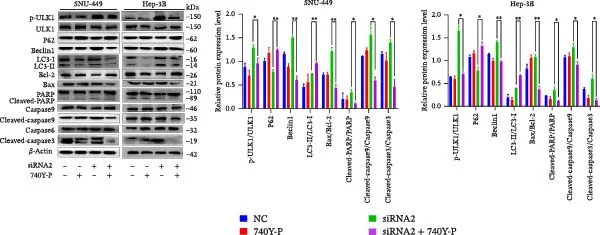
(E)

### 3.6. Downregulation of PYCR1 Induced Autophagy Dependent Apoptosis via Suppressed PI3K/AKT/mTOR Pathway in HCC Cells

**Figure 7:**
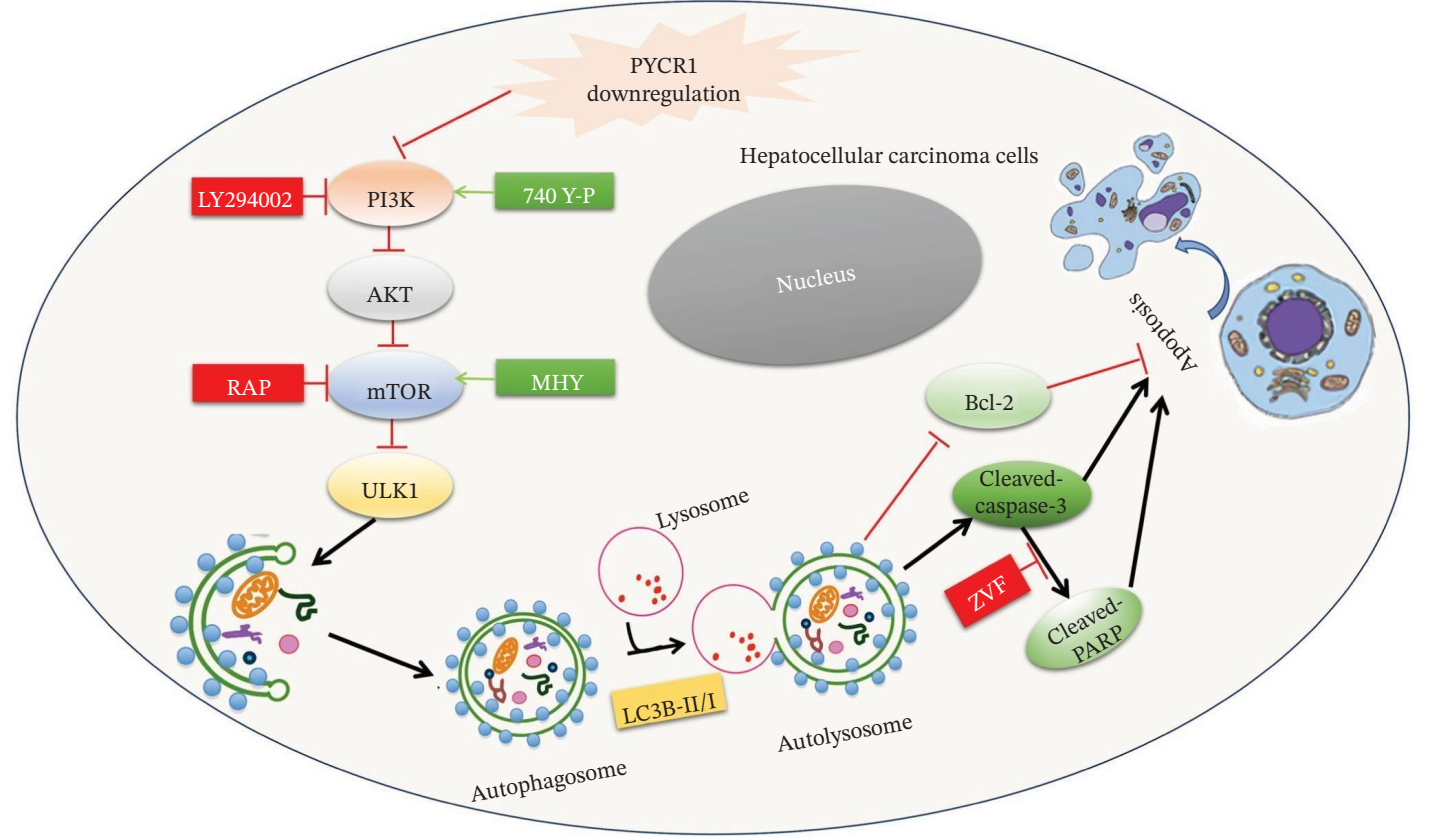
The proposed mechanistic framework of this study. PYCR1 knockdown triggered caspase‐dependent apoptosis, potentially mediated through enhanced autophagic activity, as evidenced by an increased LC3B‐II/I ratio. Notably, this pro‐autophagic effect was dependent on inhibition of the PI3K/AKT/mTOR pathway. Together, these findings demonstrate that PYCR1 suppression induces autophagy‐dependent apoptosis in HCC cells via PI3K/AKT/mTOR axis inactivation, highlighting its therapeutic potential for HCC treatment.

The PI3K/AKT/mTOR signaling pathway is one of the core pathways regulating cell growth, metabolism, survival, and death, closely related to autophagy and apoptosis. In this study, we pretreated PYCR1 downregulated cells with LY (inhibitor of PI3K) or 740 Y‐P (activator of PI3K). Compared to siRNA2‐treated cells, LY treatment markedly decreased p‐PI3K, p‐AKT1, and p‐mTOR levels, whereas 740 Y‐P substantially increased their phosphorylation (Figure 6B, C). These results demonstrate that both compounds can respectively counteract or exacerbate the suppression of PI3K/AKT/mTOR signaling induced by PYCR1 knockdown. To support the impact of this pathway in autophagy‐dependent apoptosis induced by PYCR1 downregulation, the expression of related autophagy and apoptotic proteins was detected. LY increased the LC3B‐II/I and p‐ULK1/ULK1 ratio levels induced by PYCR1 downregulation, upregulated Beclin1 expression, and reduced P62 levels. At the same time, it reduced the expression of anti‐apoptotic protein Bcl‐2 and increased the protein levels of Bax, cleaved‐PARP, cleave‐caspase9, cleaved‐caspase3, etc. (Figure 6D). In contrast, pretreatment with 740 Y‐P produced the inverse effect (Figure 6E), demonstrating that PYCR1 knockdown triggers autophagy‐dependent apoptosis through PI3K/AKT/mTOR pathway inhibition. In addition, flow cytometry and LC3 immunofluorescence staining were performed, indicating that LY or 740 Y‐P significantly increased or decreased cell apoptosis and autophagy caused by PYCR1 downregulation, consistent with previous results (Figures 5A, 6A). The above experimental results indicate that PYCR1 downregulation induces autophagy‐dependent apoptosis through inhibiting the PI3K/AKT/mTOR axis in human HCC cells (Figure 7).

## 4. Discussion

As a predominant form of primary liver cancer (85%–95% of cases), HCC continues to be a major global health burden and a leading cause of cancer mortality [23, 24]. While current treatment strategies encompass chemotherapy, radiotherapy, immunotherapy, and surgical interventions, their clinical effectiveness is frequently constrained by several limitations: extended treatment durations, elevated recurrence risks, and frequent disease advancement [25–27]. Therefore, identifying novel therapeutic targets and strategies for HCC is urgently needed. Among the myriad molecular dysregulations driving HCC, the interplay between autophagy and apoptosis has garnered significant attention as a pivotal determinant of cellular fate and therapeutic response. Autophagy can initially serve as a pro‐survival mechanism under stress but can also facilitate apoptosis under sustained or intense stimuli, a process termed autophagy‐dependent apoptosis. This axis is particularly relevant in HCC, where its modulation influences tumor progression, metastasis, and chemoresistance [28, 29]. For instance, targeting this switch has been proposed as a strategy to overcome therapeutic resistance in advanced HCC [30]. Therefore, elucidating the specific triggers and regulators of autophagy‐dependent apoptosis in HCC is of paramount importance. Our study focuses on PYCR1 precisely because its known roles in metabolism and cell survival positioned it as a potential upstream regulator of this critical switch. Nevertheless, the precise role of PYCR1 in HCC remains unclear; our data demonstrate that PYCR1 is highly expressed in HCC patients and correlates with poor prognosis. Additionally, silencing PYCR1 in HCC cells Inhibited their proliferative, migratory, and invasive capacities, consistent with previous findings [17]. These results confirm PYCR1 as an oncogenic molecule and highlight its potential as a therapeutic target for HCC.

Apoptosis, a highly controlled programed cell death process, is primarily governed by caspases [31]. As a pivotal executioner, caspase‐3 plays a central role in mediating this process [32]. Our findings reveal that PYCR1 suppression impedes HCC cell proliferation and triggers apoptosis through the upregulation of cleaved‐caspase3, cleaved‐caspase9, Bax, and cleaved‐PARP, alongside the downregulation of the anti‐apoptotic protein Bcl‐2. Interestingly, silencing PYCR1 in combination with ZVF treatment significantly attenuated apoptosis induced by PYCR1 downregulation. Autophagy serves as a critical metabolic adaptation mechanism in cancer cells, enabling them to sustain the elevated energy and biosynthetic demands of tumorigenesis. However, its role in cancer progression is context‐dependent, exhibiting both pro‐survival and growth‐suppressive effects under different conditions [7, 8]. Consequently, regulating autophagy is an attractive anticancer strategy. For instance, Qi et al. [33] showed that SHCBP1 suppresses autophagy in ovarian cancer by activating the AKT/mTOR pathway. Jin et al. [34] reported that autophagy induction inhibits cancer cell proliferation in the bladder. A key indicator of autophagy initiation is the conversion of LC3‐I to LC3‐II [35]. Here, PYCR1 knockdown substantially elevated LC3B‐II levels in HCC cells. Meanwhile, TEM and confocal microscopy confirmed an increase in autophagosome formation and LC3 protein aggregation in cells after downregulation of PYCR1, suggesting enhanced autophagic activity.

The PI3K/AKT/mTOR signaling pathway is a central regulator of cellular metabolism, proliferation, and survival, playing a pivotal role in maintaining homeostasis [12, 36]. This pathway has been implicated in autophagy modulation across multiple malignancies, including HCC [37], non‐small cell lung cancer (NSCLC) [38], and nasopharyngeal carcinoma [39]. Notably, mTOR, a serine/threonine kinase, serves as a critical node in cellular energy and metabolic regulation [13]. For instance, miR‐210 enhances autophagy in M2 macrophages via PI3K/AKT/mTOR activation, facilitating HCC progression [37]. Similarly, the chemokine CCL2 drives NSCLC metastasis and EMT through coordinated PI3K/AKT/mTOR and autophagy signaling [40]. Building on these findings, our study reveals that PYCR1 suppression triggers autophagy‐dependent apoptosis in cancer cells. Pretreatment of mTOR activator MHY before silencing the PYCR1 gene can significantly reverse the decrease in autophagosomes and apoptotic cells induced by downregulation of PYCR1. In addition, when cells are pretreated with the mTOR inhibitor RAP, this activation effect can be enhanced as autophagy volume accumulates and apoptosis increases.

The PI3K family comprises a group of enzymes ubiquitously expressed in eukaryotes [11]. PI3K catalyzes the production of PIP3, which activates AKT and subsequently enhances mTOR activity, thereby regulating critical cellular processes including metabolism, growth, and survival [12]. To investigate whether PYCR1 downregulation induces autophagy‐dependent apoptosis through PI3K/AKT/mTOR inhibition, we pretreated PYCR1‐silenced cells with either the PI3K inhibitor LY or the PI3K activator 740Y‐P. Our results demonstrated that LY treatment potentiated the suppressive impacts of PYCR1 knockdown on PI3K/AKT/mTOR pathway proteins, while simultaneously promoting autophagy and apoptosis. In contrast, 740Y‐P treatment effectively rescued the pathway inhibition caused by PYCR1 downregulation, resulting in reduced autophagy and apoptosis. Collectively, these data demonstrate that PYCR1 knockdown triggers apoptosis through an autophagy‐mediated mechanism, which appears to be partially dependent on downregulation of the PI3K/AKT/mTOR cascade. Our findings that PYCR1 knockdown inhibits the PI3K/AKT/mTOR pathway connect its function to a broader context of metabolic reprograming in HCC. Beyond its established role in controlling autophagy and apoptosis, the PI3K/AKT/mTOR axis is a central regulator of cellular lipid metabolism [41]. AKT activation promotes lipogenesis and lipid storage, processes frequently hyperactivated in HCC to support rapid tumor growth and membrane biosynthesis. Conversely, inhibition of this pathway, as observed upon PYCR1 downregulation, would be expected to disrupt these protumorigenic metabolic fluxes [42]. Recent studies underscore that dysregulated lipid metabolism is not merely a bystander but a driver of HCC pathogenesis, influencing signaling, energy balance, and the tumor micro‐environment. The AKT‐mediated lipid metabolic network is thus a crucial component of the malignant phenotype [41]. Therefore, the tumor‐suppressive effects of PYCR1 silencing may be partially mediated through a dual mechanism: direct induction of autophagy‐dependent apoptosis and indirect disruption of AKT‐driven anabolic lipid metabolism, starving the tumor cells of essential building blocks. This integrated view aligns PYCR1 with the growing paradigm of metabolic targets in cancer therapy.

## 5. Conclusion

In conclusion, this study demonstrates that PYCR1 suppression triggers autophagy‐mediated apoptosis in HCC cells via PI3K/AKT/mTOR pathway inhibition. These results position PYCR1 as a promising therapeutic target for HCC treatment strategies.

## Author Contributions

**Junli Zhang and Yachao Hou**: formal analysis, investigation, writing – original Draft. **Xinxin Jin and Biao Gu**: formal analysis, investigation. **Junli Zhang and Wenjuan Wu**: conceptualization, validation, resources, writing – review and editing, supervision, project administration, funding acquisition.

## Funding

This study was supported by the Bengbu Health Research General Project (Grant BBWK2024A207), the Anhui Province Traditional Chinese Medicine Inheritance and Innovation Research Project (Grant 2025CCCX097), and the Bengbu Central Hospital Research Project (Grant 2025bbsy16).

## Ethics Statement

The authors have nothing to report.

## Consent

The authors have nothing to report.

## Conflicts of Interest

The authors declare no conflicts of interest.

## Data Availability

The datasets used and analyzed during the current study are available from the corresponding author upon reasonable request.
